# Legal Tattoo Complicated by Sepsis and Necrotizing Fasciitis Requiring Acute Surgery

**DOI:** 10.7759/cureus.14139

**Published:** 2021-03-27

**Authors:** Simona Uggeri, Francesca Nasi, Marco Ghidoni, Fabio Gilioli, Novella Guicciardi

**Affiliations:** 1 Internal Medicine Department, Mirandola Hospital, Azienda Unita Sanitaria Locale di Modena, Mirandola, ITA; 2 Radiodiagnostic Department, Mirandola Hospital, Azienda Unita Sanitaria Locale di Modena, Mirandola, ITA

**Keywords:** tattooing, necrotizing fasciitis, sepsis, streptococcus pyogenes

## Abstract

A previously healthy 26-year-old male was referred to our hospital for fever occurring after getting a tattoo on the left leg. At the emergency department, he was promptly diagnosed with sepsis and necrotizing fasciitis of the left leg. The patient was empirically treated with vancomycin and piperacillin/tazobactam. Blood cultures identified *Streptococcus pyogenes. *Necrotizing fasciitis required acute surgery. The patient survived as a result of an early clinical diagnosis and surgical intervention.

We report the first case of sepsis and severe soft tissue infection, after legal tattooing, which required fasciotomy. All previously reported cases have been about necrotizing fasciitis in young males who received traditional Samoan tattooing, a practice usually realized with handmade tools. In our patient, the tattoo, created in Samoan style, was performed in an authorized center by a professional tattooist using contemporary sanitary techniques.

## Introduction

Tattooing is a growing trend in the world. In the medical literature, reviews have pointed out the large spectrum of adverse effects related to tattooing. Common clinical complications include sensitivity to sun and allergy to tattoo pigments. Common medical complications include infectious diseases. Fungal, parasitic, and viral pathogens may be inoculated with tattoo needle penetration. We report a case of necrotizing fasciitis seen in a young male who had received a tattoo in an authorized center.

## Case presentation

**Figure 1:**
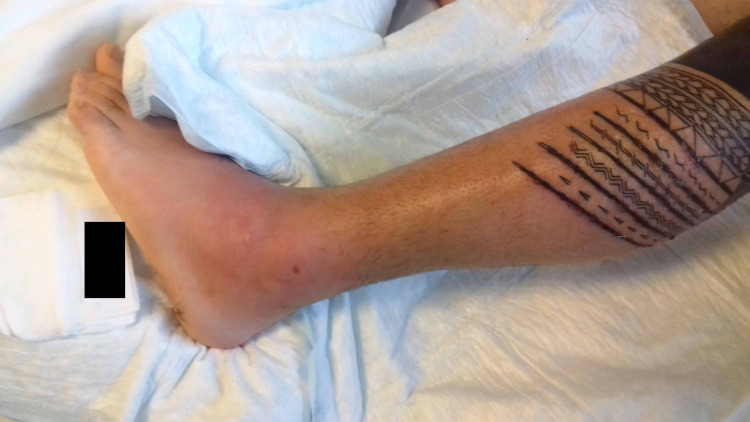
Erythema and edema along several areas of the tattooed skin.

A 26-year-old Caucasian male presented to the local emergency department with a three-day history of fever. He had no known immune disorders or underlying conditions. Fever and chills occurred approximately six hours after receiving a tattoo on the left leg. The tattoo, created in Samoan style, was performed in an authorized center by a professional tattooist using modern sanitary techniques. The tattoo covered the outer upper part of the left leg. On physical examination, erythema and edema were noted along several areas of the tattooed skin (Figure 1). He was diagnosed with sepsis secondary to soft tissue infection of his left leg.

**Figure 2:**
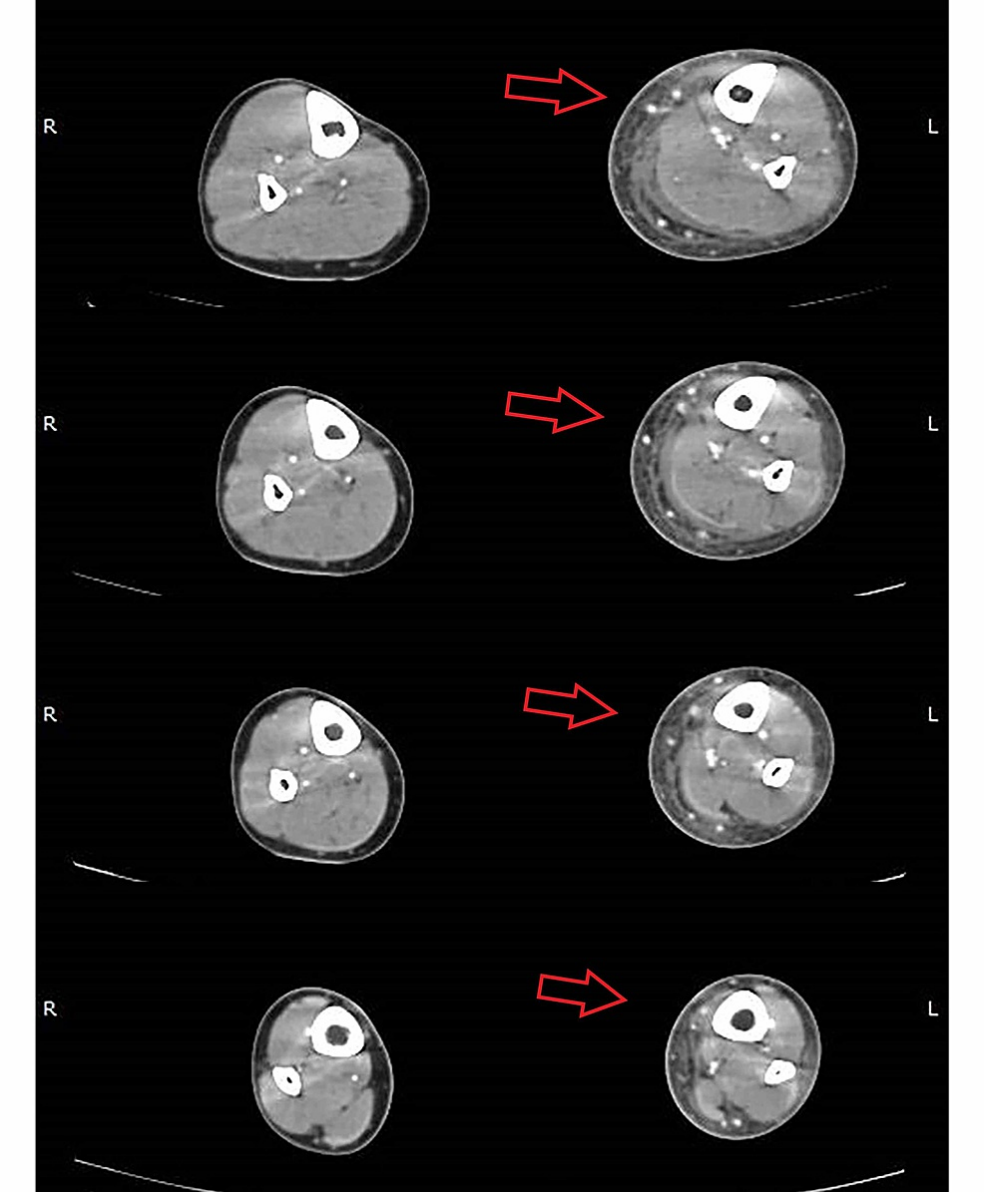
Arterial-phase CT axial images show circumferential edema of subcutaneous tissue, thickening of the crural fascia, and fluid flap on the medial side of leg, close to gastrocnemius muscle belly. CT, computed tomography

He was admitted to the internal medicine department. Fluid resuscitation was initiated. Intravenous vancomycin and piperacillin/tazobactam were empirically started. Laboratory findings showed elevated C-reactive protein (35.0 mg/dL, reference: <0.5 mg/dL) and elevated white blood cell count (20.190 × 10^9^/L, reference: 4.000-10.900 × 10^9^/L), but normal renal function, normal hepatic function, and normal serum creatine kinase levels. On the second day, computed tomography (CT) scan was performed which showed circumferential edema of subcutaneous tissue, thickening of the crural fascia, and fluid flap on the medial side of the leg (Figure 2). Blood cultures were positive for *Streptococcus pyogenes*. Clindamycin was then added to the antibiotic regimen. Mild elevation in aspartate aminotransferase (57 U/L, reference: 1-40 U/L) suggested deep infection involving muscle or fascia. The patient was always alert and hemodynamically stable, but suffering from fever and pain.

**Figure 3:**
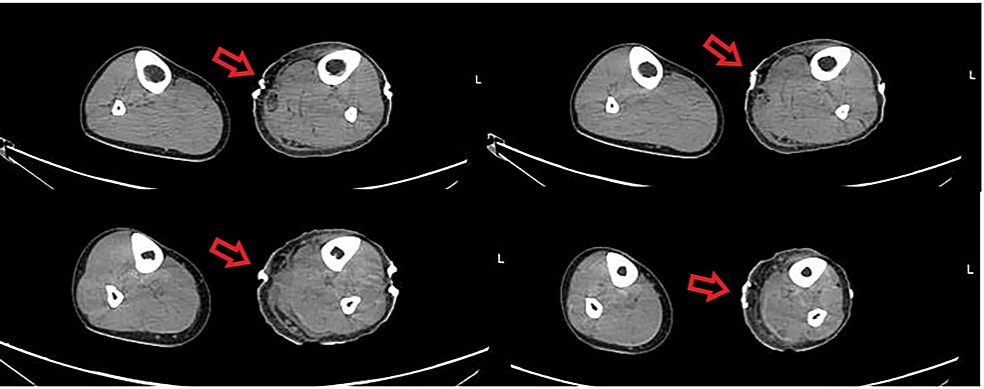
CT axial imaging: after surgery, edematous thickening of subcutaneous tissue appeared along the leg. Under the surgical metal clips, an intrafascial hydro-aerial collection is visible. CT, computed tomography

On the fourth day, he was transferred to the nearest urban hospital, and urgent fasciotomy of the left leg was performed. After surgery, the patient still reported pain and fever. CT scan showed edematous thickening of subcutaneous tissue and an intrafascial hydro-aerial collection along the leg (Figure 3). Imaging confirmed the clinical diagnosis. On the fifth day, the patient was transferred to the operating room for surgical revision where thorough debridement was performed. After surgery, he also required prolonged treatment with intravenous antibiotic therapy. After 19 days, he was discharged without any complications. One month after the discharge, the patient underwent a follow-up examination in our operative unit: he was fine and apyretic. His skin was also healing well.

## Discussion

The practice of tattooing is widespread in the world. In a comprehensive study, Kluger reviewed the epidemiology of tattooed people in Western countries. In the United States, Europe, and Australia, the prevalence of tattooing is around 10-20% [1]. It has been estimated that approximately 12% of the European population has at least one tattoo [2]. The Italian National Health Institute conducted a national survey to study the tattooed population in Italy. The prevalence of tattooed people was 12.8% of the general population in Italy. In Italy, tattooing is more frequent among women, with a prevalence of 13.8% for women and 11.7% for men. According to the data, 3.3% of tattooed people reported complications or reactions; of these, only 21.3% consulted a physician [3].

In the medical literature, reviews have pointed out a large spectrum of adverse effects related to tattooing. Common clinical complications include sensitivity to sun and allergy to tattoo pigments. Common medical complications include infectious diseases. Fungal, parasitic, and viral pathogens may be inoculated with tattoo needle penetration [4]. In rare cases, bacteria may pass in soft tissue, causing cellulitis and necrotizing fasciitis. Additionally, bacteria may enter the blood and cause sepsis, endocarditis, and cardiac insufficiency. Thus, sepsis may cause multiorgan failure and finally death [5,6].

We performed a literature search in MEDLINE (PubMed) to collect reports about sepsis and necrotizing fasciitis following traditional Samoan tattooing. Only six similar cases, described in four reports, have been published in the medical literature [6-9]. In the first report, in 1997, Korman et al. described a case of necrotizing fasciitis and sepsis with *Pseudomonas aeruginosa* and *S. pyogenes* in a 25-year-old male from Australia. The severe infection required fasciotomy. In this case, the patient had received a traditional tattoo from a Samoan tattooist. No disinfection procedures were used [8]. In 2011, McLean and D’Souza reported two young males in New Zealand with severe sepsis and acute renal failure due to soft tissue infection, which required surgical debridement. In these cases, pyogenic infection occurred after traditional Samoan tattooing [9]. In 2005, in New Zealand, Porter et al. described two young males with necrotizing fasciitis, following traditional Samoan tattooing, which required surgical debridement. In the first case, a 45-year-old male was diagnosed with septic shock secondary to necrotizing fasciitis. He underwent surgical debridement. In the second case, a 29-year-old male was diagnosed with septic shock and acute renal failure secondary to necrotizing fasciitis following traditional Samoan tattooing. He died of acute heart failure as a consequence of septic shock, as revealed by autopsy [6]. Finally, another case was published in 2013 about a 46-year-old male in the United States. He was diagnosed with necrotizing fasciitis and severe sepsis after traditional Samoan tattooing. During his hospital course, acute renal failure and myocardial infarction occurred. Soft tissue infection required surgical debridement [7].

Traditional Samoan tattooing is an ancient process, common in Polynesia and New Zealand. In Samoan traditional culture, the tattoo is a rite of passage for young men. The traditional tattoo is created by tools composed of shell, tooth, and wood [10]. The design created is usually geometric. The process consists of many sessions and takes several months. Prior to each session, the tools should be boiled in water [7]. McLean and D’Souza identified multiple problems in infection control: artists who worked in a garage or in clients’ houses, lack of running water, and the total absence of sterilization of the instruments. This study illustrated the potential risk due to traditional Samoan tattooing because this technique is often practiced in an unhygienic manner [9].

In these cases, sepsis is the consequence of traditional Samoan tattooing, a rare practice utilizing handmade tools. Instead, in this case we report necrotizing fasciitis occurring after tattooing created by a professional tattooist. Generally, modern tattoos are created by artists certified by law. In Europe, professional tattoo artists work according to modern hygienic standards. United European Tattoo Artists have written a standard protocol that guarantees the highest possible safety for both artists and customers. This protocol describes hygiene measures to be used for work areas, sterilization processes, tattoo machines and materials used, disinfection procedures, methods of storage, and use of ink [11]. Modern professional tattoo artists use an electric tattoo machine, which inserts ink into the skin via a single needle or a group of needles that are soldered onto a bar, which is attached to an oscillating unit. The unit rapidly and repeatedly drives the needles in and out of the skin, usually at a speed between 50 and 3,000 times per minute. The needles are single-use needles that are packaged individually.

Although modern techniques and recent regulations have reduced the risk of infection, complications are possible. Mistakes can occur depending on the tattooist’s skills. Negligence can occur when a tattooist fails to follow hygiene rules, placing the patient at risk of infections. The needle, while piercing the skin, introduces ink and alters the skin function, which may create an access for microorganisms. The tattoo ink itself can be contaminated during manufacture or once the bottle is opened. In other cases, contamination could occur as a result of poor hygienic conditions at the time of tattoo or due to inappropriate use of tattooing equipment, such as using the same needle or ink cap for successive clients without respecting the rules of asepsis. Moreover, microorganisms can enter into the skin in the case of inadequate disinfection of the skin area to be tattooed. Finally, after the tattoo creation, the patient can scratch the treated area and inoculate bacteria [12].

## Conclusions

Necrotizing fasciitis following tattooing is a very rare condition. To our knowledge, only six similar cases, described in four reports, have been published in the medical literature. In these reports, sepsis was the consequence of traditional Samoan tattooing, a practice usually realized with handmade tools. Traditional tattooing is often practiced in an unhygienic manner. In this case we report, differently from what has been previously reported, necrotizing fasciitis occurring after a tattoo created by a professional tattooist. Although modern techniques and recent regulations have reduced the risk of infection, complications are possible. Mistakes can occur depending on the tattooist’s skills. Negligence can occur when tattooist fails to follow hygiene rules, placing the patient at risk of infections.

In conclusion, modern tattooing is a form of minor surgery performed by non-physician workers, and may place the patient at risk of severe infections. Necrotizing fasciitis following tattooing is a rare condition. This report aims to draw the attention of emergency medicine physicians and clinicians to this life-threatening but treatable condition.
